# Variola and Vaccine

**Published:** 1892-03

**Authors:** 


### VARIOLA AND VACCINE.

Dr. Chauveau, Professor in the Musem of Natural History of Paris, communicated to the public a *résumé* of his researches on the subject of the transformation of the virus of small-pox into vaccinia, or vaccine :—

It is well known that a number of physicians and most of the people believe that vaccine, or vaccinia, produced from the bovine species for the purpose of vaccination against small-pox, is nothing else than small-pox virus itself, modified by successive passages through the animal organism. The Commission of Lyons, France, under the direction of Dr. Chauveau, has established, it seems very positively, that (as has long been believed by most thinking scientists) this is not the case. The virus used for vaccination (cow-pox or horse-pox) is certainly a close relative of the virus of small-pox, and it is perhaps legitimate to admit that they were derived from a common source or from another, in centuries past. But at this time they are a distinct species, and it seems impossible to reduce them to a single species by the known methods and artifices of experimentation. Inoculations of small-pox in the horse and in cattle produce variola in them, and the introduction of the virus thus produced in these animals, gives to man variola itself. It was found that the animals inoculated with small-pox became immune against vaccinia, just the same as man inoculated with vaccinia becomes immune against variola.

The symptoms of these different viruses in animals vary considerably, and there can be no mistake made in the diagnosis.

Messrs. Haccius and Eternod of the Vaccinal Institute of Lancy, Geneva, produce a vaccinal lymph cultivated on calf, which is said to be transformed from variola, and not very long ago published their conclusion that variola and vaccinia are probably identical. M. Chauveau, in experimenting with this lymph, found that it contained, in reality, the true virus of small-pox, and therefore it is a dangerous article to use for vaccination. Vaccine, then, should not for practical purposes be considered as an attenuated variola virus, for the two are now distinct, and both are strong viruses of their respective species, whatever their origin may have been.—*The Bacteriological World and Modern Medicine*, *Jan.*, *1892*.

